# Non-Contact Analysis of the Adsorptive Ink Capacity of Nano Silica Pigments on a Printing Coating Base

**DOI:** 10.1371/journal.pone.0109918

**Published:** 2014-10-16

**Authors:** Bo Jiang, Yu Dong Huang

**Affiliations:** Polymer Materials and Engineering Department, School of Chemical Engineering and Technology, Harbin Institute of Technology, Harbin, People's Republic of China; Washington State University, United States of America

## Abstract

Near infrared spectra combined with partial least squares were proposed as a means of non-contact analysis of the adsorptive ink capacity of recording coating materials in ink jet printing. First, the recording coating materials were prepared based on nano silica pigments. 80 samples of the recording coating materials were selected to develop the calibration of adsorptive ink capacity against ink adsorption (g/m^2^). The model developed predicted samples in the validation set with r^2^  = 0.80 and SEP  = 1.108, analytical results showed that near infrared spectra had significant potential for the adsorption of ink capacity on the recording coating. The influence of factors such as recording coating thickness, mass ratio silica: binder-polyvinyl alcohol and the solution concentration on the adsorptive ink capacity were studied. With the help of the near infrared spectra, the adsorptive ink capacity of a recording coating material can be rapidly controlled.

## Introduction

With the development computers and digital techniques, ink-jet printing has been widely used in many applications, such as organic chemistry [1]–[4], electronics [5], [6], nanotechnology [7], [8], [9], and tissue engineering, etc. The recording coating in the ink jet printing (RC-IJP) affects the printing quality. The adsorptive ink capacity of the recording coating is evaluated by the per unit area adsorptive ink weight, it is an important factor in ink jet printing. If the per unit area recording coating weight is invariant (uniform), an excess of ink causes diffusion of the ink on the recording coating, while a sub-optimal amount of ink influences the ink absorption and leads to poor quality in printing.

Nano-silica is widely applied as pigment particle in recording coating. Its size, distribution and the specific surface area influences ink adsorption. Big specific surface area causes the strong adsorption capacity; the dispersibility of the recording coating is more uniform in terms of the more adsorptive ink. Polyvinyl alcohol is generally used as binders on recording coating. Its molecular structure influences the absorption effect too. The high molecular weight of polyvinyl alcohol leads to a close molecular structure, this structure causes fastness on adsorptive ink drop and excellent water-resistance; small molecular weight causes loose structures, which lead to easy ink impregnation and clear colors. These results influence the clarity and permanent properties of the image. In order to enhance the printing quality, the analysis of the adsorptive ink capacity of RC-IJP is very important. Further, rapid analysis of the adsorptive ink capacity has never been reported.

Near infrared (NIR) spectroscopy is a very rapid, accurate and non-destructive method for simultaneous measurements on different constituents in various products [10]–[12]. Nowadays, the traditional method is human eyeballing for the adsorptive ink capacity of RC-IJP. However, this non-technical method is associated with large deviations. The aim of this study was to develop a non-destructive and rapid NIR spectroscopy method for the prediction and control of the adsorptive ink capacity of RC-IJP.

## Experimental

### 1. Preparation of the recording coating

The inorganic pigments-nano scale silica with the average nanoparticle size of 12 nm, and the specific surface area of 188 m^2^/g was obtained from Degussa A200, Germany. In addition, the binder-polyvinyl alcohol (PVA), acroart and accessory ingredient is provided from Shanghai Chemical Co., Ltd, China.

The silicane dispersing agent (gamma-aminopropyltriethoxysilane) is added into distilled water with magnetic stirring for 10 min. Nitric acid is subsequently added until solution pH = 4. Silica is added in the above solution under homogenizer (Fluko Equipment Shanghai Co., Ltd.) for 30 min. Solution concentration is 10%, mass ratio between silicane dispersing agent and silica is 0.02∶1. Above preparation product is added in 8% PVA solution by the homogenizer for 10 min (mass ratio SiO_2_∶PVA = 3∶1), then, the mixed solution is placed in the oven at 40°C for 120 min. Consequently, the recording coating is prepared. The blade coater method is used in the preparation of recording coating materials, this process is shown in **Materials S1**.

### 2. Instrument analysis

The surface morphologies and roughness of RC-IJP wereobserved using a high-resolution scanning electron microscopy (SEM), energy dispersive spectrum (EDS) (Quanta 200F, made in the USA) and atomic force microscope (AFM, Solver-P47H, NT-MDT, Russia). NIR spectra are collected by a diffuse reflection FT-NIR spectrometer (MATRIX, Bruker, Germany). Spectra are collected using contactless way, the light from the sources is focused on to RC-IJP (The distance is 17 cm between light source and samples), and then the diffuse reflectance spectra from the RC-IJP are recorded by the spectrometer.

### 3 Reference analysis of adsorptive ink weight

**Figure 1:**
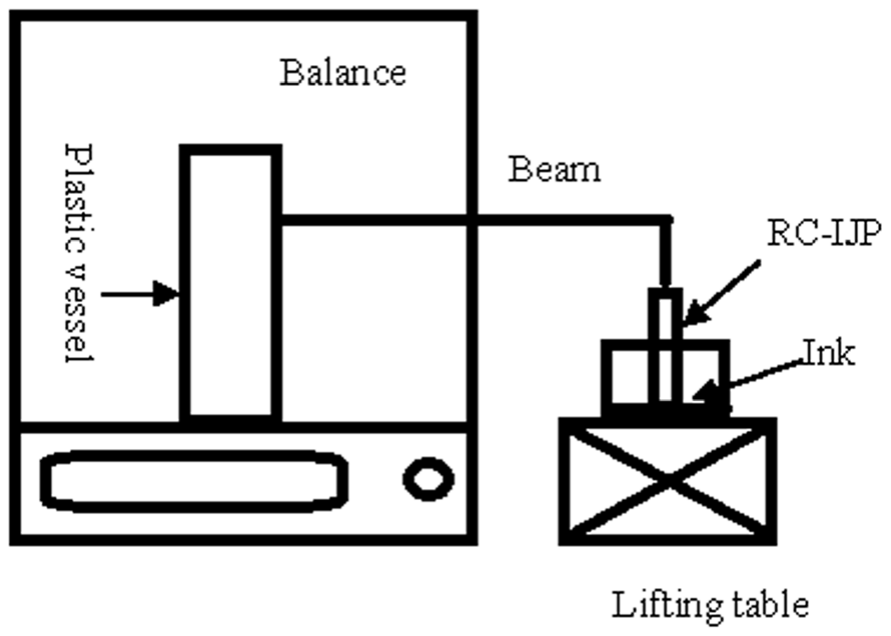
Equipment of adsorptive ink weight of RC-IJP.

Equipment of adsorptive ink weight of RC-IJP is shown in Figure 1. Plastic vessel is placed on balance. RC-IJP is hanged by beam. End of RC-IJP contacts the ink. The adsorptive ink weight is shown by a weighing balance (Precision is 0.0001 g). The adsorptive ink capacity is evaluated by the per unit area adsorptive ink weight in RC-IJP.

## Results and Discussion

### 1. Analysis of the adsorptive ink property of RC-IJP

**Figure 2:**
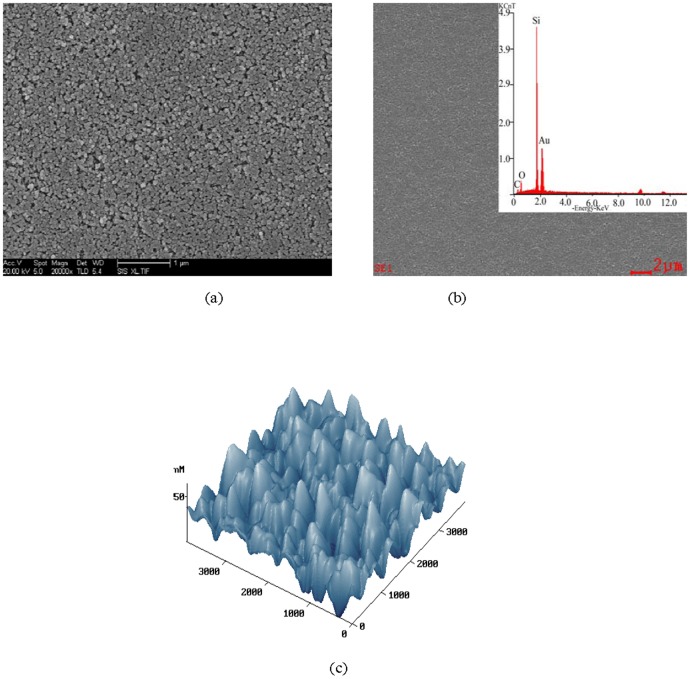
Structure and infiltration analysis of RC-IJP: (a) SEM (b) EDS (c) AFM.

The micro-structure of RC-IJP is analyzed by SEM. It can be observed from Figure 2 (a) that the dispersibility of the recording coating is uniform. The excellent dispersibility is beneficial to infiltration of the ink. If weight of per unit area is invariant, the dispersibility of the recording coating is more uniform with the use use of more adsorptive ink. In order to further analyze the microstructure of RC-IJP, the main element composes C, O, Si and Au on recording coating is studied by EDS in Figure 2 (b). Samples are spurted gold in the aluminum piece, in order to enhance electroconductivity in the analytical process of samples. So, Au element is shown in EDS. AFM images of the recording coating are provided in Figure 2(c). From Figure 2 (c), it can also be observed that the surface of the recording coating has roughness, which is beneficial to adsorption of the ink.

### 2. Theoretical basis of NIR spectrum and adsorptive ink capacity analysis

As the NIR-spectra showed information of the presence of hydrogen-containing groups, the recording coating mainly contained SiO_2_, PVA and dispersing agent, these components contain the hydrogen-containing groups. So, NIR spectra can analyze the RC-IJP information

**Figure 3:**
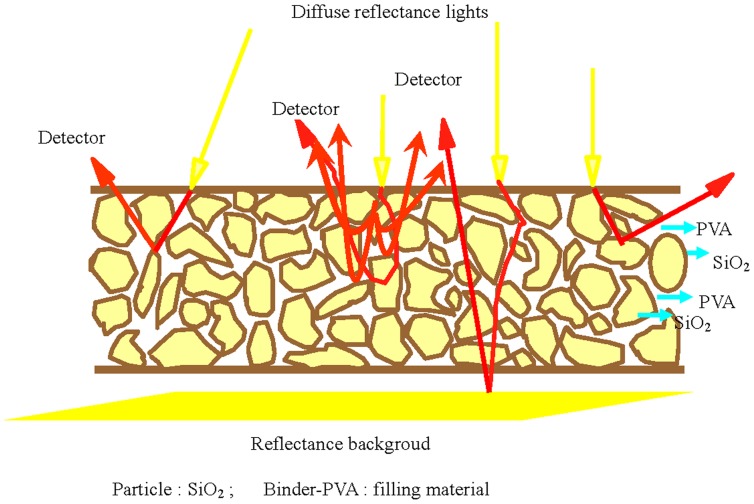
Mode of action NIR diffuse reflectance light on RC-IJP.

The mode of action NIR diffuses reflectance light on RC-IJP is shown in Figure 3. Near-infrared diffuses reflection light focus on surface samples, then light undergoes much reflection into samples and returns to surface samples. Finally, reflected light with sample information is absorbed by detector. So, SiO_2_ and PVA component contents, molecular structure and particle distribution influences the Near-infrared diffuse reflection light absorption degree. Above influence factors *C _j_* provide following equation:
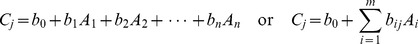
(1)


*C _j_*: Influence factors;

*b_0_*: Constant in equation;

*b_ij_*: Coefficient in equation;

*A_i_*: Linear function value in *i* wavelength location.

The quantitative relation is found between influence factors and the NIR spectra feature of the samples by statistical methods.

The adsorptive ink capacity is confirmed by the recording coating. Nano-silica particle size, distribution and the specific surface area influences ink adsorption. Big specific surface area causes the strong adsorption capacity; the dispersibility of the recording coating is more uniform in terms of the more adsorptive ink. Binder-polyvinyl alcohol molecular structure influences the absorption effect also. High molecular weight lead to the molecular structure of the close, this structure causes fastness on adsorptive ink drop and excellent water-resistance; small molecular weight is loose structure, which leads to ink impregnation easy and colors clear.

### 3. Information of RC-IJP NIR Spectrum

**Figure 4:**
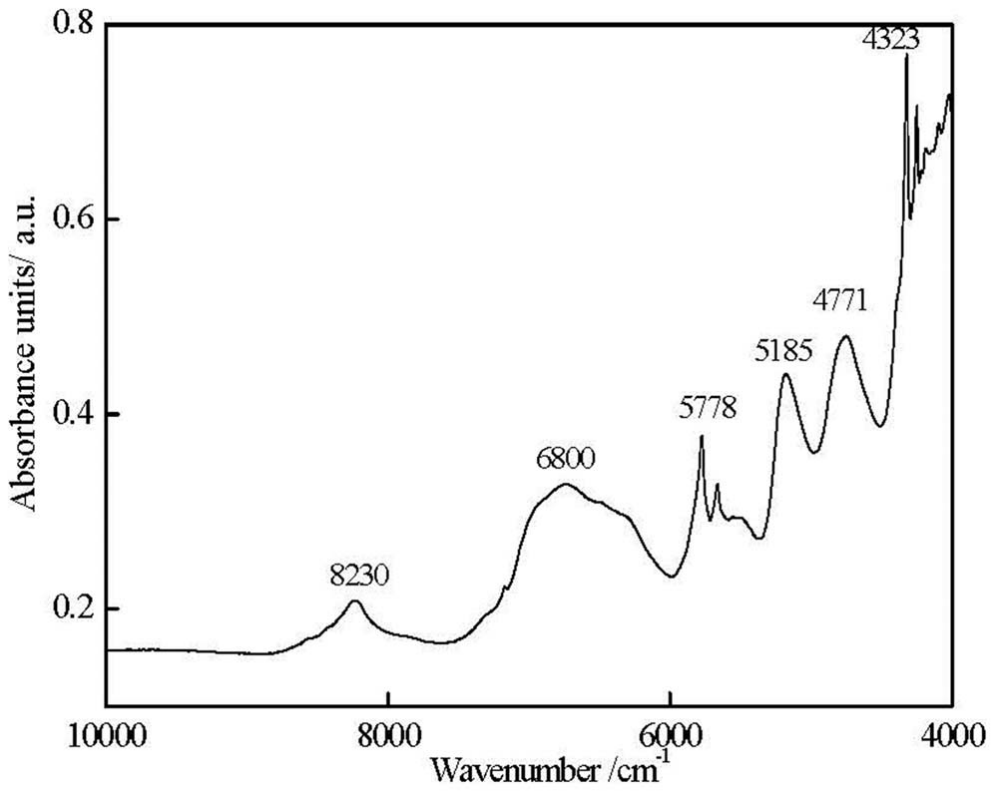
NIR diffuse reflectance spectral of RC-IJP.

Above analysis showed that NIR spectroscopy enables the analysis of the adsorptive ink capacity of RC-IJP. RC-IJP spectra are collected by a diffuse reflection FT-NIR spectrometer in Figure 4 (NIR spectrometer is operated in the NIR region from 4000 to 12000 cm^−1^. Each spectrum is obtained by an average of 8 scans with a resolution of 16 cm^−1^). FT-NIR information of the spectrum is given: the methyl groups and methene groups produced combination bands at 4323 cm^−1^ and 4250 cm^−1^, the methyl groups gives an overtone band at 5778 cm^−1^, the methene groups gives second overtone bands at 8230 cm^−1^, respectively. In addition, ethenyl demonstrates combination bands at 4772 cm^−1^. On the other hand, hydroxy gives stretching combination and overtone bands at 5185 cm^−1^and 6800 cm^−1^. The above analysis adequately represents the required information of the RC-IJP structure in NIR spectra.

### 3. The calibration model and optimization of the RC-IJP adsorptive ink weight

The determination coefficient (*R^2^*), root mean square error of calibration (*RMSEC*) and root mean square error of prediction (*RMSEP*) were calculated to evaluate the model to find the best calibration model. The linearity correlation coefficient is a measure of the consistency between the NIR prediction values and actual values for the calibration sets. In developing model, 97 samples (80 calibration samples, 17 predication samples. The NIR spectra of 97 samples are shown in **Materials S1**) of RC-IJP are used. Reference method of the adsorptive ink weight is analyzed by developing equipment.

**Figure 5:**
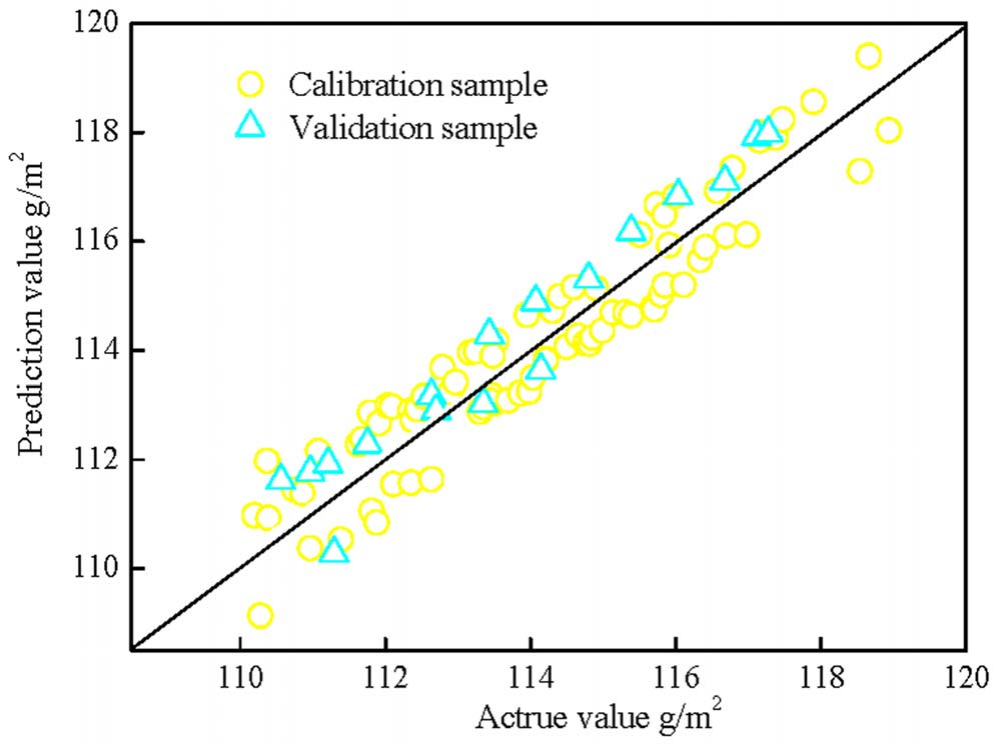
Correlation between predicted values and actual values of adsorptive ink weight of RC-IJP.

**Figure 6:**
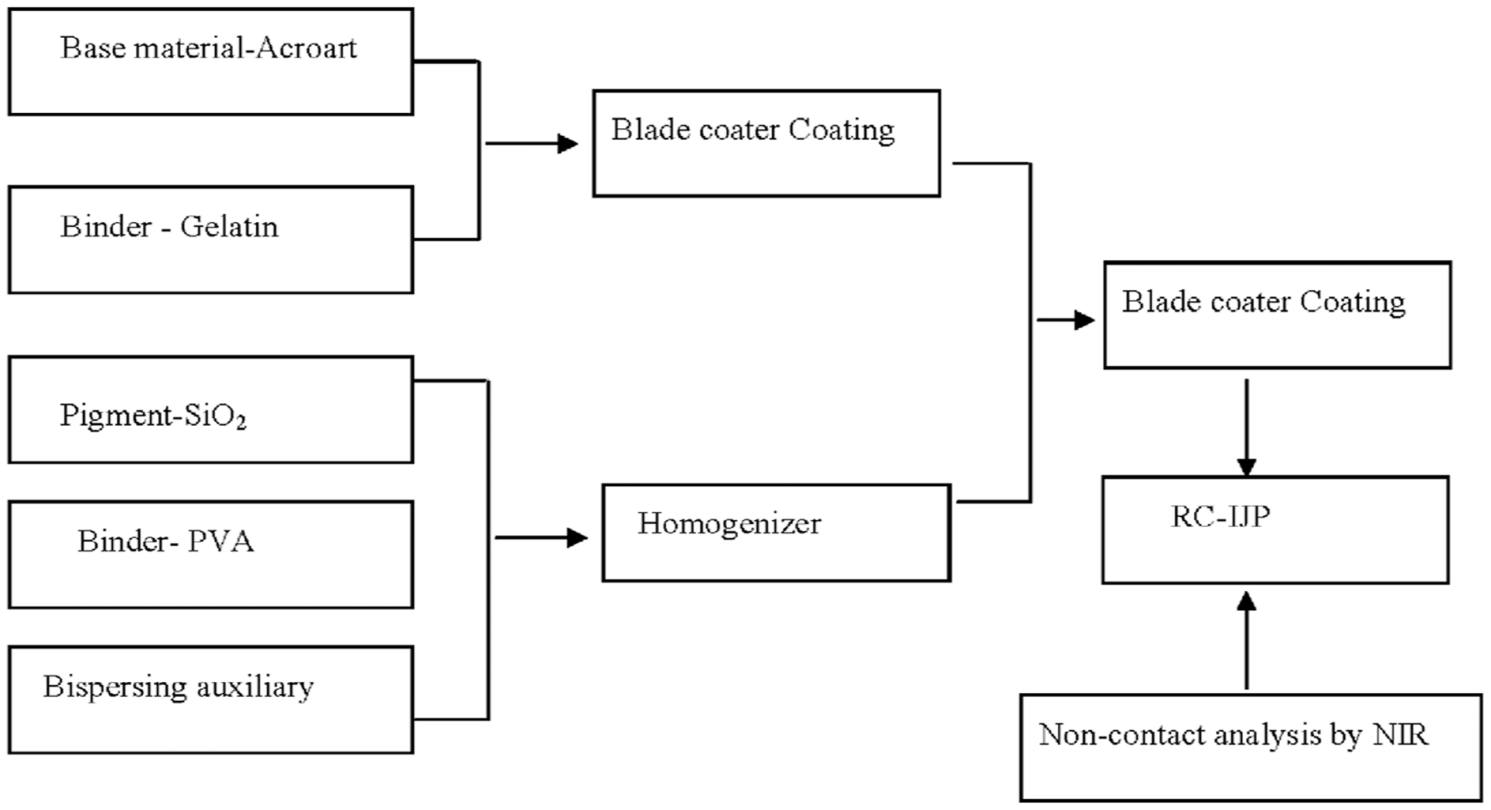
Sketch of RC-IJP analysis by NIR spectra.

Table 1 shows the parameter of the adsorptive ink weight of the developing model. 80 samples are developed for the calibration model. The maximum and minimum values are 110.14 g/m^2^ and 118.84 g/m^2^ for per unit area adsorptive ink weight, respectively. The Calibration model is developed by PLS, and full cross-validation is applied for optimization. Min-Max normalization preprocessing methods gave the best values for *R^2^* (0.82), *RMSEC* (0.896) and *RMSEP* (1.108), respectively. The spectral ranges obtained the best wavenumber of 4000–5800 and 6700–9000 cm^−1^, 5 PLS factors are used. The plots of the actual values versus the predicted values by NIR for calibration set of the adsorptive ink weight were shown in Figure.5. The regression coefficient of the calibration model is 0.94. 17 prediction samples were used, the plot of the actual values against NIR predicted values for the prediction sets was shown in Figure.5 also. From the above analysis, NIR method is a good alternative technique for analyzing the adsorptive ink weight in RC-IJP, the sketch of RC-IJP analysis by NIR spectra is shown in Figure.6.

**Table 1:** Statistical results of the developing models.

Quality indexes	Range	Pre-processing routines	Spectral range (cm^−1^)	PLS factors	R^2^	RMSEC	RMSEP
Adsorptive ink weight	110.14–118.84 (g/m^2^)	Min–Max normalization	4000–5800 6700–9000	5	0.82	0.896	1.108

**Figure 7:**
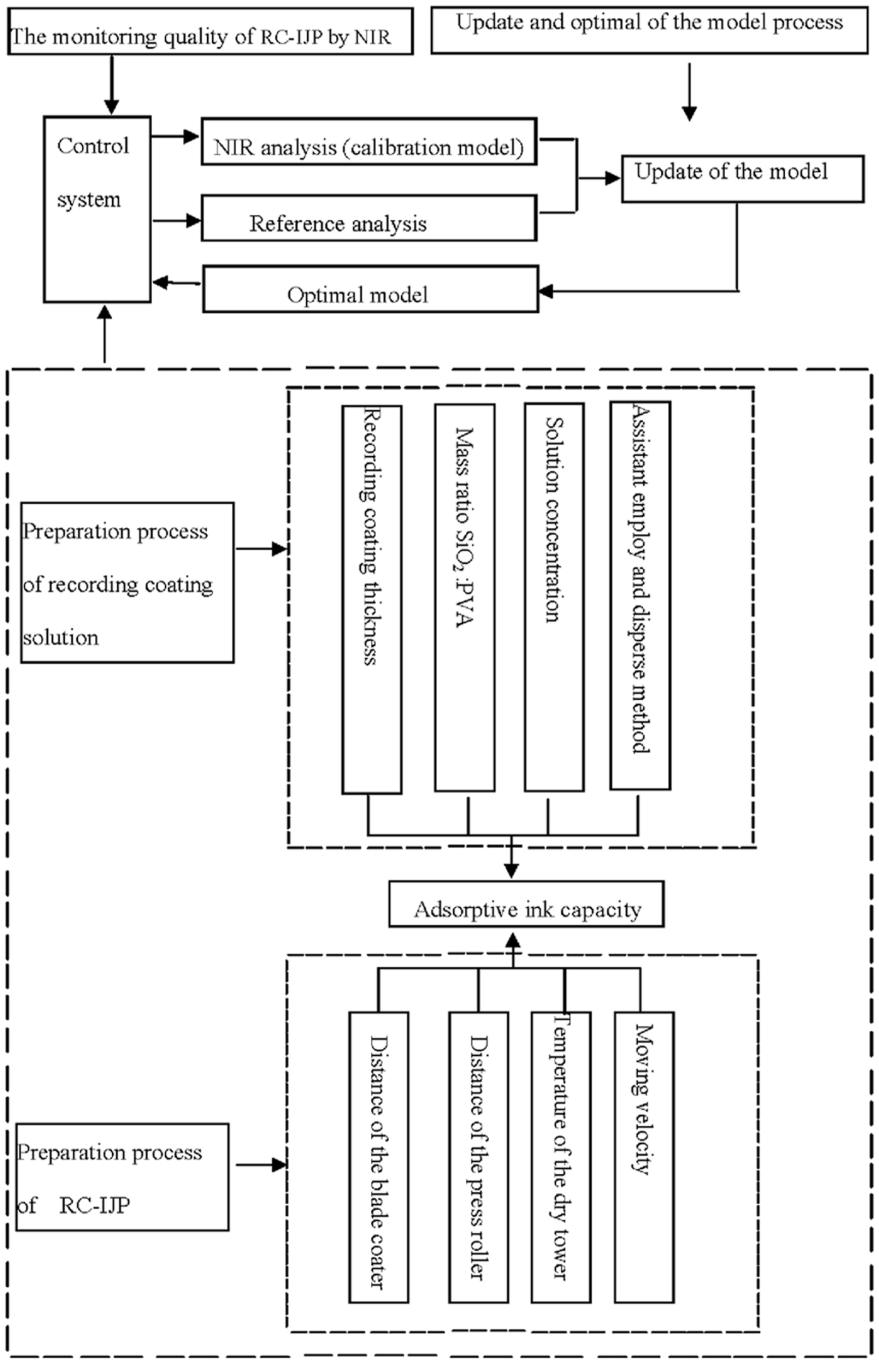
Sketch of monitoring quality of RC-IJP.

If the processing parameters and conditions are changed, the calibration models would be updated. New samples are selected from the production line and added to the calibration model again. After updating and optimization, the calibration models are developed again. The developing models are implanted to the control system, the processing parameters are adjusted rapidly by NIR spectroscopy. With the help of NIR spectroscopy, RC-IJP adsorptive ink capacity is controlled timely. The preparation processing parameters of the recording coating are adjusted based on recording coating thickness, mass ratio SiO_2_∶PVA, solution concentration and disperse method. On the other hand, the distance of the blade coater or press roller, the temperature of the dry tower, the moving velocity can also control the adsorptive ink capacity in preparation of RC-IJP. It is worth taking into consideration that co-adjustments of all the parameters can be complicated. we have only focused on the main parameters in this study although other factors such as the quality of the acroart, environmental humidity, temperature etc., can also affect the RC-IJP quality. Above process is shown in Figure.7.

## Conclusion

From the above analyses, the adsorptive ink weight of the recording coating was successfully performed based on NIR spectra. With the help of NIR spectroscopy, RC-IJP quality is controlled timely. In addition, from the analysis of the adsorptive ink weight, it was observed that when per unit area weight is invariant; the dispersibility of the recording coating is more uniform in terms of the more adsorptive ink.

## Supporting Information

Materials S1 **The blade coater method is used in the preparation of recording coating materials, this process can be found in the Supporting Information, and NIR spectra of 97 samples (80 calibration samples, 17 predication samples) of RC-IJP are shown in Materials S1.**
(DOC)

## References

[1] Chae SS, Min H, Lee JH (2013) Fabrication of a Multidomain and ultrafast-switching liquid crystal alignment layer using contact printing with a poly(dimethylsiloxane) stamp. Adv. Mater. 25: 1408––1414. 23280963 10.1002/adma.201202927

[2] Kim YH, Yoo BJ, Anthony E, Park SK (2012) Controlled deposition of a high-performance small-molecule organic single-crystal transistor array by direct ink-jet printing. Adv. Mater 24: 497–502. 22213548 10.1002/adma.201103032

[3] de Gans BJ, Xue L, Agarwal US, Schubert US (2007) Inkjet printing of luminescent CdTe nanocrystal-polymer composites. Adv Funct Mater 17: 23–28.

[4] de Gans BJ, Duineveld PC, Schubert US (2004) Inkjet printing of polymers: state of the art and future developments,. Adv Mater 16: 203–213.

[5] Yoshioka Y, Jabbour GE (2006) Inkjet printing of oxidants for patterning of nanometer-thick conducting polymer electrodes. Adv Mater 18: 1307–1312.

[6] Sirringhaus H, Kawase T, Friend RH, Shimoda T, Inbasekaran M, et al (2000) High-resolution inkjet printing of all-polymer transistor circuits. Science 290: 2123–2126. 11118142 10.1126/science.290.5499.2123

[7] Lee KJ, Jun BH, Kim TH, Joung J (2006) Direct synthesis and inkjetting of silver nanocrystals toward printed electronics. Nanotechnology 17: 2424–2428.

[8] Wu JT, Hsu SLC, Tsai MH (2012) Direct ink-jet printing of silver nitrate-silver nanowire hybrid inks to fabricate silver conductive lines. J.Mater.Chem 22: 15599–15605.

[9] Chung J, Ko S, Bieri NR, Grigoropoulos CP, Poulikakos D (2004) Conductor microstructures by laser curing of printed gold nanoparticle ink. Appl Phys Lett 84: 801–803.

[10] Fernandes DDS, Gomes AA, Costa GB, Silva GW, Véras G (2011) Determination of biodiesel content in biodiesel/diesel blends using NIR and visible spectroscopy with variable selection. Talanta 87: 30–34. 22099644 10.1016/j.talanta.2011.09.025

[11] Moraes IR, Kalbac M, Dmitriev E, Dunsch L (2011) Charging of self-doped poly(anilineboronic acid) films studied by in situ ESR/UV/Vis/NIR spectro electro chemistry and ex Situ FTIR Spectroscopy. Chemphyschem 12: 2920–2924. 21990178 10.1002/cphc.201100567

[12] Jiang B, Huang YD (2008) Noncontact analysis of the fiber weight per unit area in prepreg by near-infrared spectroscopy. Anal Chim Acta 616: 103–108. 18471490 10.1016/j.aca.2008.04.013

