# Supplementary material for: Non-Contact Analysis of the Adsorptive Ink Capacity of Nano Silica Pigments on a Printing Coating Base
Source: PLoS One. 2014 Oct 16;9(10):e109918. doi: 10.1371/journal.pone.0109918 (PMC4199666; doi:10.1371/journal.pone.0109918)
Supplement: Materials S1 — The blade coater method is used in the preparation of recording coating materials, this process can be found in the Supporting Information, and NIR spectra of 97 samples (80 calibration samples, 17 predication samples) of RC-IJP are shown in Materials S1. (DOC) [file pone.0109918.s001.doc]

Materials S1

Non-contact analysis of the adsorptive ink capacity of nano silica pigments on a printing coating base

**Bo. Jiang*, Yu. Dong. Huang**

Polymer Materials and Engineering Department, School of Chemical Engineering and Technology, Harbin Institute of Technology, Harbin, People’s Republic of China

**The preparation of RC-IJP by the blade coater method**

The blade coater method is used in the preparation of RC-IJP. In order to enhance the property of polarity on acroart, the surface is disposed by corona, and then primer is coated on acroart. The excessive solution is removed off by the blade coater. In order to evaporate the solvent, acroart with gelatin is moved through a dry tower. In the next step of process, the recording coating is coated on acroart with gelatin. RC-IJP is wrapped to the take up mechanism. Preparation schema is shown in *Figure S1*.

**Instrument analysis**

NIR spectra of 97 samples (80 calibration samples, 17 predication samples) of RC-IJP are shown in *Figure S2*.

Solution recoverer

Corona

Blade coater

RC-IJP

Dry tower


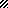


Take up mechanism

Acroart


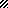


Gelatin


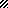


Dry tower

Solution recoverer

RC

Blade coater

*Figure S1.* Schematic of preparation process of RC-IJP


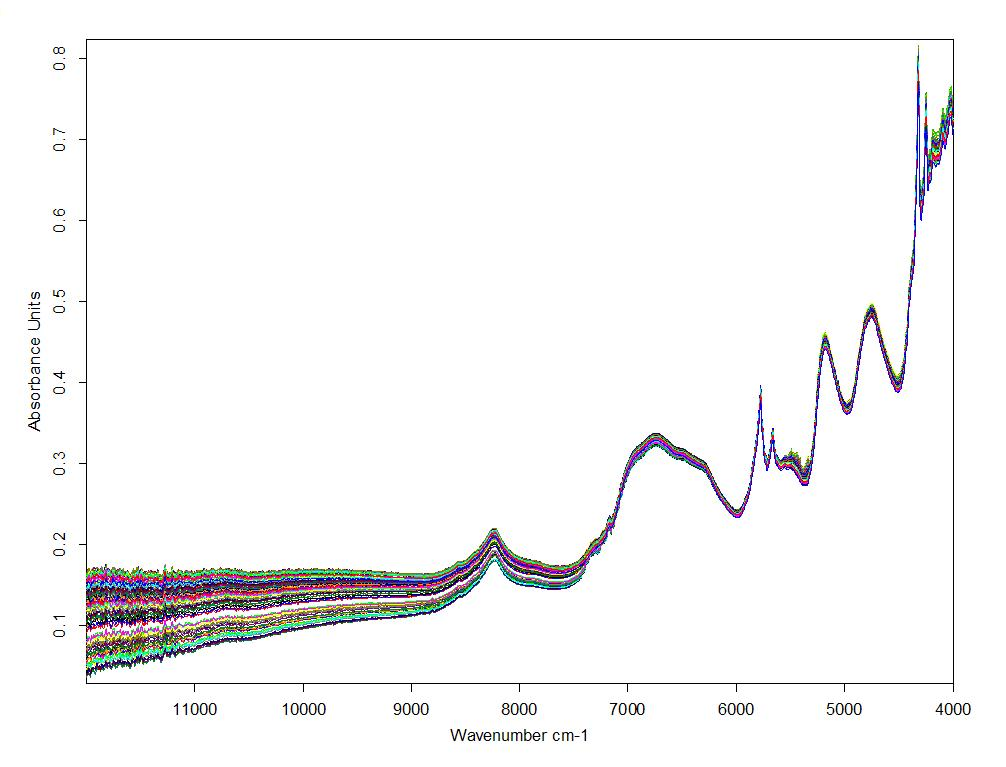


*Figure S2* : NIR spectra of 97 samples
